# Biopollution by Invasive Marine Non-Indigenous Species: A Review of Potential Adverse Ecological Effects in a Changing Climate

**DOI:** 10.3390/ijerph18084268

**Published:** 2021-04-17

**Authors:** Anna Occhipinti-Ambrogi

**Affiliations:** Department of Earth and Environmental Sciences, University of Pavia, Via Sant’Epifanio, 14, 27100 Pavia, Italy; anna.occhipinti@unipv.it; Tel.: +39-0382-984-876

**Keywords:** biological invasions, risk assessment, Mediterranean Sea, pollution management

## Abstract

Biopollution by alien species is considered one of the main threats to environmental health. The marine environment, traditionally less studied than inland domains, has been the object of recent work that is reviewed here. Increasing scientific evidence has been accumulated worldwide on ecosystem deterioration induced by the development of massive non-indigenous population outbreaks in many coastal sites. Biopollution assessment procedures have been proposed, adopting criteria already used for xenochemical compounds, adjusting them to deal with alien species invasions. On the other hand, prevention and mitigation measures to reduce biopollution impact cannot always mimic the emission countermeasures that have been successfully applied for chemical pollutants. Nevertheless, in order to design comprehensive water-quality criteria, risk assessment and management strategies, based on scientific knowledge, have been developed in a similar way as for chemical pollution. The Mediterranean Sea is a well-known case of alien species invasion, mainly linked to the opening of the Suez Canal. Non-indigenous species have caused well-documented changes in many coastal ecosystems, favoured by concomitant changes induced by global warming and by the heavy load of nutrients and pollutants by various anthropogenic activities. Naval commercial traffic and leisure boats are among the most active vectors of spread for alien species inside the Mediterranean, and also towards other ocean regions. The scientific evidence gathered and summarized in this review suggests that effective management actions, under a precautionary approach, should be put in place in order to control introductions of species in new areas. These management measures are already established in international treaties and national legislations, but should be enforced to prevent the disruption of the dynamic ecological equilibria in the receiving environment and to control the direct adverse effects of alien species.

## 1. Introduction

The idea of translating definitions and concepts used in marine pollution assessment and control into the field regarding the growing concern about alien species (AS) was clearly formulated by [1]. In the context of marine studies, biological pollution caused by the introduction of non-native species is considered one of the main threats to the environmental health of the oceans [2,3]. The introduction of AS into the marine environment is a factor of disturbance that can be consistently viewed as a pollution agent, albeit of a different nature if compared with physical and chemical factors. As far as AS are concerned, comparatively few scientific contributions have been devoted to the marine environment, while the terrestrial and inland water domains have been treated in more detail [4,5]; yet, sufficient scientific evidence has been accumulated, starting from the foundations delivered by [6,7]. The actual and potential ecosystem deterioration induced by the development of massive non-indigenous population outbreaks has been documented in various coastal sites (e.g., [4,8,9]), and surveys of alien species introductions are actively performed, encompassing all of the continents [10]; see also for Europe [11,12]; for the Americas [13,14,15,16]; for Africa [17,18,19]; for Asia [20,21,22,23]; for Oceania [24,25]; and for the Arctic and Antarctica [26,27].

Biopollution, while having a distinct ontology and phenology from chemical pollution, can be assessed and evaluated [28,29,30] following some of the paradigms that have been widely used for xenochemical compounds, albeit with some adaptations that must be used to deal with xenobiotic organisms. In particular, the effects of the often sudden population outbreaks by species introduced from separate biogeographic marine regions are not completely amenable to dose–effect responses used in traditional toxicological assessments of chemical pollutants.

Nevertheless, biological introductions do have implications for the environmental quality of estuarine and coastal areas. As with chemical pollutants, consequences can be detected at the individual level (as in the case of attacks by parasites or pathogens), or at the population (by genetic change, i.e., hybridization), or at the community level (by structural shifts). Habitat (by modification of physical–chemical conditions) and ecosystem changes (by alteration of energy flow and organic material cycling) are also possible. Some biopollution assessment methods have been proposed [2,31] in order to classify the impact on natural ecosystems, while measures of the more complex assessment of economic impact are rare [32,33]. Functional consequences of IAS (invasive alien species) along coasts and within estuarine systems have not been explored extensively, yet published studies show that species addictions affect ecosystem functioning (e.g., productivity, biogeochemical cycles, and decomposition) and biotic relations (e.g., prey–predator interactions, and introductions of parasites and diseases). These effects will contribute to ecosystem functioning in complex ways and range from almost negligible to dramatic proportions [34,35].

This paper will review (i) the definitions proposed to study biopollution caused by the introduction of AS; (ii) some of the impacts that have been described in the literature; and (iii) the framework of risk assessment procedures that have been applied aiming at management of biological invasions. Moreover, (iv) the Mediterranean Sea, known as one of the world’s hotspots for biological invasions, offers good examples of biopollution; and (v) climate change interacts with biopollution, potentially determining an unprecedented phase shift in the global distribution of marine species. In the last section, a few considerations on biopollution management in the marine realm are proposed.

The literature review has been compiled with the aim of illustrating some points of contact and synergy between the approaches used in the study of biological invasions and of pollution, searching scientific literature databases such as Google Scholar and Scopus using the key words “introduced/alien/nonindigenous/nonnative species” and “biological invasion/biopollution/biosecurity”, combined with the terms of the different paragraphs, which are “Impact”, “Risk assessment”, “Mediterranean Sea”, “Climate change”, “Management”, and “Policy”. The most recent and relevant papers were selected in order to illustrate the main features of this vast domain and to provide a primer for environmental students and practitioners to deal with this raising item of concern for conservation and welfare.

## 2. Definitions

The distinction between “biological introductions” and “biological invasions” is an important one, because the two terms are often used interchangeably. Not all introductions of AS result in large effects such as those experienced by bioinvasions. Moreover, an invasion may be caused by either natural or human activities, while an introduction is always human mediated, either intentionally or unintentionally. The sharp increase in the frequency, magnitude, and geographic span of biological invasions in the last 50 years is almost entirely due to human activities, both in general terms [36,37,38] and in the marine domain [6,39].

Here, a widely accepted definition of non-indigenous species (NIS) (synonyms: alien, exotic, nonnative, allochthonous, and introduced) has been adopted. They are species, or lower taxa, occurring outside their natural range (past or present) and dispersal potential. This includes any part, gamete, or propagule of such species that might survive and subsequently reproduce [4,40,41,42]. Their presence in the given region is either due to an intentional or unintentional introduction, resulting from human activities, or due to their arrival without human assistance from an area to which they are alien. Human action transports species across environmental barriers that include land masses or hydrographic separation of open ocean spaces. It should be noted that in the case of climate altering the distribution of species (see below), these should not be qualified as NIS, unless there is evidence that these species have been spread by humans (see [33]). A species is defined as cryptogenic [43,44], when it cannot be reliably demonstrated as being either introduced or native (because of insufficient taxonomic knowledge or due to a lack of records [43,44]).

The number of records of aquatic NIS is steadily growing since the last 50 years: first records of detection (1965–2015) across 49 aquatic ecosystems worldwide have been censused [45]. Patterns of detection rate, richness, and transport pathways revealed an annual mean of 43 (±16 SD) primary detections of NIS. The global rate of detections increased rapidly after 1995, peaking at 66 primary detections per year during 2005–2010 and then declining marginally. Detection rates were variable within and across regions through time. Arthropods, molluscs, and fish were the most frequently reported groups. Most NIS were likely introduced as stowaways in ships’ ballast water or as biofouling [45,46].

Invasive alien species (IAS) are a subset of established NIS that have spread, are spreading, or have demonstrated to be able to spread elsewhere, causing adverse effects within invaded regions [47]. Cryptogenic species may demonstrate invasive characteristics and so may be included in IAS assessments. It should be noted that invasive species that cause harmful effects are not necessarily alien. Native organisms can also attain high levels of abundance and so interfere with human activities, impacting the environment or modifying local biodiversity.

The knowledge-base on NIS continuously expands and many databases are collecting information on NIS. Currently, there are more than 250 websites on NIS, along different geographical ranges (a few examples are reported in Table 1).

Besides inventories of NIS, information on NIS origin, introduction history, pathways, vectors, actual distribution, and more are provided by most databases. The databases have been used increasingly for scientific analyses, though key information required for bioinvasion management and research are only partially reported [5].

Biopollution is a dynamic process: for instance, the spread of a species within a biogeographical region after having been introduced from elsewhere has raised special interest. In this case, the primary inoculation consists of an arrival from a distant source. Secondary spread is defined as the transport to new regions within the same biogeographical area following establishment [40]. Secondary spread may disperse alien biota more efficiently and so could compromise the ability to manage the invasion [33].

Biological pollution consists of the negative effects of AS on environmental quality, at the level of organism, population, community, habitat, or ecosystem [1,28]; therefore, AS are considered to be biological contaminants [48]. They may also cause damage to human health [49,50] and/or to economic activities, so their presence must not be underestimated [48]. Their establishment and spread seem directly linked to propagule pressure, which has been recognised as the major factor in the success of an invasion and depends on the quality, quantity, and frequency of invaders [51,52].

The environmental status of marine waters is traditionally being evaluated taking into account the effects of various forms of chemical pollution, eutrophication, habitat destruction and overexploitation. Biopollution should also be considered in environmental assessments, as stated in the European Marine Framework Strategy Directive. In fact, listing the Good Environmental Status descriptors, the Directive declares that ‘‘Non-indigenous species introduced by human activities are at levels that do not adversely alter the ecosystem’’ [53,54].

## 3. Impacts by NIS

Adverse effects of AS (especially invasive species) on ecosystems have been shown as biodiversity loss, alteration of food webs, physical habitat disruption, and import of parasites and diseases [37,39,55,56,57,58,59,60,61,62]. Some examples of the ecological and environmental impacts of non-indigenous marine species have been listed by [63]. A progressive homogenization of biota [64] across regions and continents, as a consequence of increased translocation of species, is raising concern among ecology students [65,66].

While information on the total number of non-indigenous species, their traits, invasiveness potential, and vectors of spread has accumulated in many parts of the world, scientific evidence is still generally scanty to draw general conclusions on the real magnitude of their ecological impact [67,68,69,70]. In addition, ecological functions can change dramatically over time, or else changes can become evident only after long periods of innocuous presence of the alien species. Such cryptic processes may lead to an underestimation of the long-term impacts and constrain the effectiveness of management actions [71].

Communities of organisms can change over historical (ecological) time in three ways: species can be deleted (extinctions), added (invasions), or can change in relative abundance. In marine environments, while the latter two types of alterations are increasingly recognized (if not extensively studied), extinctions in historical time have received little recognition [72,73,74]. This lack of attention to marine extinctions stands in striking contrast to the comparatively advanced recognition of extinctions in terrestrial ecosystems [75].

In order to describe the relevance of the impacts caused by alien species, many different approaches have been proposed in the literature: a selection of these different approaches, both concerning individual species and assemblages of NIS, is briefly outlined below.

The IUCN’s [76] Environment Impact Classification for Alien Taxa (EICAT) is being proposed as a standard for categorizing alien species’ impact. It is used to classify individual alien taxa according to the magnitude of their impacts on native taxa, based on the organizational level in the affected community (https://www.iucn.org/theme/species/our-work/invasive-species/eicat, accessed on 4 April 2021). Impact categories range from Minimal Concern to Massive. If only individual performance is affected, it is considered a Minor impact; if a native taxon is removed from the community (locally extinct or extirpated), it is considered Major or Massive, based on the reversibility of the change [76,77]. The IUCN EICAT Standard is the product of a long process of developing and adapting frameworks to quantify impacts. One evolution is SEICAT [61], a standardized method for classifying alien taxa in terms of the magnitude of their impacts on human wellbeing, based on the capability approach from welfare economics. The core characteristic of this approach is that it uses changes in people’s activities as a common metric for evaluating impacts on wellbeing.

The INvasive Species Effects Assessment Tool (INSEAT) also contributes to the current scenario of invasive species assessment. INSEAT considers both positive and negative impacts of IAS on ecosystem services (ES) and uses the ES framework, commonly classified into provisioning, regulating, and cultural services. This differs from SEICAT, which uses the constituents of human wellbeing, and from EICAT, which defines its own categories of environmental impacts [78].

Considering the overall pressure of AS introduced into a new habitat is a matter of concern for environmental management, Vandekerkhove et al. [79] have underlined that the current assessment measures used to classify Environmental Status are seldom considering the impact of invasive aquatic species. Nevertheless, managers can make use of different indexing tools that have been designed in order to measure the (at least potential) impact of invasive alien species on marine ecosystems.

Olenin et al. [2] developed an index that classifies the impacts of NIS on marine native species, communities, habitats, and ecosystem functioning. Their method has been repeatedly used to evaluate impact (using five different levels of biopollution [80]) and has proved useful to classify within existing schemes for water quality assessment (phytoplankton in the Baltic Sea [81]; benthos in Baltic German estuaries [82]; macroalgae in Ireland [83]). Both spatial and temporal comparisons have been performed. The assessments have been useful to evaluate the performance of various measures designed to mitigate impacts and to prevent further introductions [84].

Çinar and Bakir [85] developed ALEX, a biotic index based on the relative importance of AS within benthic communities, these latter were categorized as casual (group II), established (group III), and invasive species (group IV). The index produces scores that categorize the benthic quality status from bad to high, indicating the magnitude of the cumulative effects of alien species. This metric discriminated stations according to different levels of degradation, therefore confirming variability across a gradient of human-mediated impacts. The ALEX index was also used in different situations [86,87,88,89].

Katsanevakis et al. [90] proposed the Cumulative Impact of Invasive Alien Species (CIMPAL), based on the distribution of invasive species and of the affected areas. Mazaris and Katsanevakis [91] calculated the cumulative impact scores in the Natura 2000 sites of the Mediterranean Sea, highlighting that the database of the Natura 2000 sites was lacking the basic information about this important threat to protected areas.

Structured protocols can help reduce biases and improve accuracy and transparency, and discussions can help resolve disagreements among assessors. The assessment protocols developed to evaluate the current and potential impacts of AS have not yet gained a general consensus, partly because consistency among them has proven unsatisfactory [92]. To effectively tackle the challenge of biological invasions through targeted strategies and mitigation measures, managers and policy makers require adequate reporting and flow of information. A promising approach has been presented by the project InvasiBES—understanding and managing the Impacts of INVASIve alien species on Biodiversity and Ecosystem Services (http://elabs.ebd.csic.es/web/invasibes, accessed on 4 April 2021)—that aims to provide a comprehensive understanding of the multi-faceted impacts of biological invasions on biodiversity and ecosystem services by synthesising knowledge across habitats (terrestrial, freshwater, and marine) and scales (continental to local) [93].

In discussing the concept of biopollution itself, one should also be aware of some tendencies creeping along the biological literature, invoking a sort of invasive species denialism [94,95,96]. Of course, the lack of a large and convincing evidence base on NIS invasion impacts is an element that reinforces this way of thinking [97]. A particular aspect of denialism (often gladly invoked by interested actors in the market) aims at emphasizing the positive effects of some introductions of AS in given environments, in order to enhance depleted biological production, or even to replace native species gone locally extinct due to other causes [98,99,100,101]. In general, the claim of increasing community biodiversity by adding new species [102] is gaining momentum. Following this claim, even the intentional introduction of non-indigenous species could be promoted or, at least, the so called “naturalized” species should not be included within the indicators of bad water quality, and active eradication measures should not be taken (see [103]).

## 4. Risk Assessment

Adverse effects of invasive alien species (IAS), or biological pollution, as discussed in the previous section, is an increasing problem in marine coastal waters and is getting the attention of managers and decision makers [46,104,105]. The need for assessing the size of the problem and the decision to put in place effective mechanisms to prevent introductions—as well as to contain, control or eradicate the introduced species that may cause damages—are more-and-more felt as an imperative. The ever-increasing movement of goods and materials through the world’s oceans makes the consequences of the global transfer of species a growing cause of concern. However, it is very difficult to predict which of NIS introductions may result in detrimental effects on environmental quality by way of changes to the biological, chemical, and/or physical properties of an invaded ecosystem [51].

Risk assessment [106,107,108,109] and management strategies [58] have been in many instances derived from the practice of chemical pollutants. Moreover, water quality criteria have encompassed both biological, physical, and chemical conditions of the environment in many legislations based on scientific knowledge [47,110,111,112,113,114,115]. Srėbalienė et al. [109] compared risk assessment frameworks developed to support the implementation of the international Ballast Water Management Convention [116] and European Regulation on Invasive Alien Species [53]. Based on their analysis of different methods used to implement the two frameworks, they found that all methods incorporated at least some general information about NIS under consideration, such as taxonomic identity and scope of the Risk Assessment [109]. The Risk Assessment components concerning reproduction and spread, pathways, stages of invasion process, distribution, and impacts were incorporated within most methods. The least covered components were the estimated consequences of economic damage and any known uses and benefits. In conclusion, the categories of impacts on human health and economy were underrepresented compared with impacts on the environment.

The Aquatic Species Invasiveness Screening Kit (AS-ISK)—which is available for free download at www.cefas.co.uk/nns/tools (accessed on 4 April 2021)—has been designed to identify potential IAS with respect to the risk assessment area. Described in detail in Copp et al. [117], the AS-ISK is fully compliant with the “minimum standards” [118] for the assessment of AS for the European Commission Regulation on the prevention and management of the introduction and spread of invasive alien species [47]. The AS-ISK consists of 55 questions: the first 49 questions (Basic Risk Assessment—BRA) cover the biogeography/historical and biology/ecology aspects of the species under assessment, including risks of introduction, establishment, dispersal, and impact. The other six questions require the assessor to predict how future climatic conditions (Climate Change Assessment—CCA) are likely to affect the BRA. In the recently released AS-ISK v2, which the assessors employed in their study, the 16 taxonomic groups of aquatic organisms previously accounted for in AS-ISK v1 [117] have been expanded to a total of 27, following the classification of living organisms by Ruggiero et al. [119]. The risk-screening index has been largely used in the US—despite some criticism of the need of expert opinion, subjectivity of climate matching, and need for regional calibration [120]—and also in the marine and freshwater context [121,122].

An additional possibility to develop a procedure for risk analysis could follow the INSEAT (INvasive Species Effects Assessment Tool) framework, developed by Martinez-Cillero [78]. INSEAT adopts a bidirectional scoring system to quantify ecosystem service gains and losses caused by alien species and could easily be adapted to evaluate risks by alien species the marine environment.

Molecular and genetic tools are made available in order to refine and strengthen the identification and the origin of biological invaders, thus giving a better ground for risk analysis and biosecurity assessments [123,124,125,126]. Two main techniques are gaining success in marine invasion ecology: DNA barcoding of individual species and metabarcoding of species assemblages. DNA barcoding is an easy-to-use, cost-effective, and standard approach, useful to validate species identification based on morphological traits. Metabarcoding introduces the possibility of nontargeted surveillance of molecular biodiversity in whole communities by extracting and barcoding either bulk DNA from environmental samples or environmental DNA [127]. Though only some groups of aquatic NIS are detectable based on the available data, the current availability of sequences of taxa with environmental and/or economic impact continues to increase with time [123,128]. Insight on the invasion history of the Japanese amphipod *Grandidierella japonica* Stephensen 1938 has been obtained, e.g., by Pilgrim et al. [129], combining information on the distribution data with molecular genetic data. Collection data showed that many bays and estuaries of the Pacific North American coast had been colonized. DNA barcode sequence data for *G. japonica* revealed two distinct clades in different localities: the presence of cryptic diversity within the species, suggesting distinct introduction events, showed the contribution of genetic analyses to the ecological understanding of colonization. An example of analysis by a molecular tool such as metabarcoding has been provided by von Ammon et al. [125]. They investigated cryptic organisms, difficult to distinguish by conventional morphological taxonomy in samples of marine biofouling, improving the detection of non-indigenous species.

## 5. The Special Case of the Mediterranean Sea

The Mediterranean Sea has been the theatre of an unprecedented wave of introduced species [90,130].

Changes in the Mediterranean marine biodiversity related to introduction of NIS have been reported as the consequence of intense maritime traffic, opening of artificial channels [131,132], and aquaculture activities [133,134]. The species richness of marine organisms in the Mediterranean Sea is estimated to be ~17,000 taxa [135], among which over 600 are NIS that have developed stable populations [136,137], from phytoplankton to fish. Numbers of NIS in the Mediterranean basin are continually evolving, as checklists of NIS are subjected to frequent changes due to better identification of taxa, new data from molecular analyses and new phylogenetic studies. Only 10% of all NIS in the Mediterranean can be considered as invasive, or potentially invasive [70].

The high number of NIS is linked in large part to the opening of the Suez Canal and to its subsequent enlargement and deepening [132], which in turn has caused well-documented changes in many coastal ecosystems [130,138], especially in the eastern part of the basin. The consequences of such a pressure of introduced species are gradually extending to the north-western sub-basins [136], favoured by concomitant changes induced by global warming and by the heavy load of nutrients and pollutant deriving by an intense anthropogenic pressure by a dense coastal development.

Special hubs for NIS introduction and development have been recognised in a few locales also outside the Levantine basin, such as the lagoon of Venice [101,139,140], the Taranto inner basins—Mar Grande e Mar Piccolo [52], the Sicily Strait [141,142], the Thau lagoon [143], and the Aegean Sea [144].

Naval traffic [145] and recreational boats [146,147] are among the most active vectors of spread for alien species. Moreover, the Mediterranean Sea has been the source for further expansion of IAS to other regional seas outside Gibraltar, thanks to the intense traffic through the strait. A few examples will illustrate the variety and magnitude of biopollution in the Mediterranean Sea.

A special and spectacular case is constituted by the green macroalgae of the genus *Caulerpa* J.V. Lamouroux, 1809 [148]. The spread of *Caulerpa cylindracea* Sonder, 1845, is one of the most threatening invasions in the Mediterranean Sea. This species, of Australian origin, was introduced into the Mediterranean by unknown vectors: perhaps by shipping (ballast waters) or due to accidental escape from an aquarium. It is now widespread and frequent in the basin [149]. Many correlative and experimental studies have focused on different aspects of *C. cylindracea* invasion. Piazzi et al. [150] evaluated the main factors influencing the spread of this alga through an overview of the results from 47 published papers on this topic. They proposed a conceptual model synthesizing the main biotic and abiotic factors that influence *C. cylindracea* spread. The mechanisms of *C. cylindracea* success and of its impacts have been recently investigated by studies on the biogeochemistry of the sediments and on the interactions of this invader with native species, microbial processes and deposition dynamics (see [151]).

The ctenophore *Mnemiopsis leidyi* A. Agassiz, 1865, is yet another “cult species” among Mediterranean NIS. It originates from the American west coasts and has given rise to blooms in the plankton of different areas of the Mediterranean, since its invasion in the Black Sea in the early 1980s [152]. The presence of this gelatinous zooplankton species may have a strong impact on zooplankton community function as it has been observed in the Black Sea [153]. Besides these effects, both *M. leidyi* and its predator *Beroe ovata* Bruguière, 1789, exert complex effects on the nutrient concentrations and all lower trophic level components of the marine ecosystem [8]. Excretion and mucus secretion have an impact on the chemical variables of the environment and on the structural and functional traits of the lower food-web communities of the Black Sea ecosystem (bacteria, pico-phytoplankton, nano-autotrophic/heterotrophic flagellates, micro-phytoplankton, chlorophyll a, primary production, and micro-zooplankton). A recent set of observations from a French lagoon, part of the Camargue brackish water natural system [154], has highlighted the extreme variability of population dynamics. The records of *M. leidyi* at temperatures ranging from 3 to 29 °C and salinity from 5 to 25 are consistent with the eurythermal and euryhaline tolerances of this ctenophore. In similarly variable physical conditions, *M. leidyi* has been present along the French lagoon ecosystems since at least 2005 (in the Berre Lagoon [155]), and probably earlier.

A localized, yet very rapid case of invasion occurred on the south side of the Strait of Gibraltar, in Ceuta (northern Africa), where the exotic seaweed *Rugulopteryx okamurae* (E.Y. Dawson) I.K. Hwang, W.J. Lee and H.S. Kim (2009) was detected in 2015 [156]. Within one year, *R. okamurae* demonstrated an extraordinarily high competitive capacity and growth. More than 5000 tons of biomass was extracted from beaches in Ceuta, and it has since spread relentlessly on rocky bottoms of the subtidal zone up to a 40 m maximum depth. The high coverage (80–90%) of *R. okamurae* is having a severe impact on local benthic communities that were displaced. A submarine monitoring station installed in 2013 on a shady substrate in the El Estrecho Natural Park, on the north side of the Strait of Gibraltar (Tarifa), detected the presence of *R. okamurae* in July 2016 and recorded the subsequent increase in coverage. The *R. okamurae* range expanded subsequently in the south of the Iberian Peninsula, towards the Atlantic (2018) and the Mediterranean coasts (2019). This bloom could have been associated with the temperature peak in July 2015 and was thus possibly linked to global warming [157].

At a larger scale, the temporal dynamics of fish perceived as “new” or increasing in different fishing areas of the Mediterranean has been investigated by Azzurro et al. [158], by using the so called “local ecological knowledge (LEK)”. They interviewed over 500 small-scale and recreational anglers across 95 locations and nine different countries, and collected semi-quantitative information on yearly changes in species abundance. Overall, 75 species were mentioned by the respondents, mostly warm-adapted species of both native and exotic origin. Respondents belonging to the same biogeographic sectors described coherent spatial and temporal patterns and revealed gradients along latitudinal and longitudinal axes.

## 6. Prospects of Biopollution in a Warming Ocean Scenario

Impacts by biopollution cannot be adequately described without considering climate change. Climate change will interact with many other human impacts to produce effects greater than with climate change alone. Together they will impact ecosystem goods and services [159,160].

Climate change is accompanied by unprecedented rates of between-continent dispersal events, associated with ever-increasing levels of international trade and human travelling [161,162]. The combined effects of climate and rapid transport are likely to bring worldwide changes to the earth’s biota, which could easily exceed the impact of either climate change or invasion acting on its own. Indeed, many of the species that will become major components of the new globalized biological communities are already present, either as rare naturalized species or in artificial manmade habitats [163,164,165]. In protected areas, the risk of invasion also needs to be quantified under current and projected climate conditions. Prioritization of key vectors and vulnerable areas enable development of effective management strategies [166].

Seawater warming has been associated with both the northward expansion of species and their increasing abundances [167,168,169]. Many studies provided evidence for the causal relationship between temperature, species distribution, and abundance [170,171,172]. Some empirical studies have specifically linked climate change to the increasing abundances of non-native species, with reference to marine systems (e.g., [173]). Previous studies indicate that temperature increases similar to those predicted by climate change models will have a strong impact on marine species [174,175], but less is known about the responses of marine invaders relative to native species [176,177].

The ability of the invasive *Mnemiopsis leidyi* to establish worldwide, both at present and in the future in the case of global warming prolongation, has been estimated by [178]. Model assessments predict accelerated Sea Surface Temperature (SST) rise in the Northern Hemisphere. As a result, waters previously too cold for *M. leidyi* reproduction could become favourable, which is especially true off the Arctic coasts of Eurasia. These areas in summer and autumn are expected to be much wider and extend closer to the North Pole. It is important to point out that all *M. leidyi* habitats, including the native, recipient, and prospective ones, are situated in shallow coastal areas and inland water bodies (semi-closed and closed seas, bays, lagoons, and fjords). Equally important is that the local currents can provide only the relatively short distance of *M. leidyi* transport, while shipping acts as the major vector. Other modelling approaches to predict NIS population developments in a changing climate have been used for benthic organisms [179].

Global warming promotes the expansion of NIS towards higher latitudes and increases the risks of introductions originating from warmer climate regions [176,180], for example by means of ballast water transfer. The role of different pathways and vectors may also shift due to climate change, alteration in environmental quality, political and social events, management policy, and the emergence of new trading routes. Intensification of human activities in sub-Arctic and Arctic areas, including a seasonal opening of Northern Sea Routes, is likely to increase the risk of new bioinvasions [46]. These processes may cause significant changes in the marine biodiversity on both coasts of the North Atlantic Ocean [51].

Increasing temperature, acceleration of sea-level rise, changes in freshwater runoff, and increasing hurricane intensity are among the climate forcings that will affect coastal ecosystems. These forcings interact with each other and with human impacts. They should be taken into account while considering the global change of marine species distribution under the influence of human activities [181]. For example, sea-level rise and lower freshwater inflow lead to both increased salinity and longer flooding duration, resulting in multiple stresses on coastal environments that are prone to the introduction of non-native species and possibly favour their massive development.

The sustainability of social and economic development must be based on the functioning of natural ecosystems. The coupled effects of climatic change and biopollution must be incorporated into environmental planning. The economic development in the coastal zone should be based on ecological integrity, because natural resources of the coastal zone represent a valuable capital that supports the economic health of society. The goods and services provided by natural capital represent the interest generated by human investment in protecting ecosystems. The perspective of sustainable development should be linked to the ecological integrity of coastal ecosystems with healthy and resilient conditions [182].

The changes, which are taking place across many different taxa and through different regions of the globe, have significant implications for biodiversity, ecosystems, and society [183], and are considered to be particularly apparent in the Mediterranean, a semi-enclosed sea, which is warming faster than any other marine region in the world [184,185,186].

## 7. Policy and Management Issues

The management of alien species introductions, already contemplated in international treaties and national legislations [187], should be enforced, notwithstanding the difficulties arising from the global nature of the phenomena and the difficulty posed by the continuity of ocean waters, as experienced before with chemical compounds across national borders.

As highlighted by Elliott [1], there are many aspects in which introduced biological organisms can be regarded as being no different from chemical pollutants. In comparing the approach used by traditional and innovative strategies put in place to protect the marine environment, it might be of interest to have a comparative look in relation to established paradigms used for chemical pollutants and the attempts made to tackle the threat posed by the biological pollution of NIS. Bellas et al. [188] observed that a new challenge in marine pollution monitoring is based (inter alia) on the harmonization of two European Union directives for the protection of the marine environment, namely, the Water Framework Directive [189] and the Marine Strategy Framework Directive [53]. Although implemented unevenly through the Union [190], both Directives consider NIS introduction as one of the criteria to establish the environmental status of water bodies. As stated by Bellas et al. [188], these two Directives were constructed following either a risk assessment approach (WFD) or an ecosystem approach (MSFD). The latter in fact established a legislative context demanding the use of effect-based tools for pollution assessment.

A promising approach to summarize large datasets of complex data in integrative indices, and to simplify the interpretation for stakeholders and decision makers, has been recently proposed by Regoli et al. [191]. Their quantitative Weight of Evidence (WOE) model (Sediqualsoft) supports a comprehensive process of “site-oriented” management decisions. A further integration with biological pollution aspects could lead to a generalized framework for strengthening the current environmental legislation that aims to preserve and protect marine ecosystems more effectively, and promoting their sustainable use.

Integrated approach schemes are needed to improve our understanding of the concomitant, and often interrelated, factors of ecosystem damage. A crucial knowledge gap is also the elaboration of internationally agreed assessment criteria for both environmental pollutants and biological responses. Knowledge about the environmental behaviour and ecotoxicity of pollutants, as well as about the ecological determinants of AS introduction and their impact on native communities, is particularly relevant for integrative monitoring purposes, together with the development of new approaches and technologies in marine pollution monitoring.

A comprehensive parallel analysis of terms used in chemical and biological pollution had been designed by Elliot [1]; a simplified list of analogous key characteristics is proposed in Table 2, especially considering the features emerged in recent times. Each discipline, i.e., chemical and biological pollution, made important forward steps, although in quite an independent way, and an integrated perspective is now necessary to deal with environmental threats.

To identify future challenges and opportunities facing invasion science, a systematic approach known as Horizon Scanning can be applied [192]. It is used for exploring emerging trends, issues, opportunities, threats, and events that can facilitate proactive responses by scientists, managers, and policy makers. Through consensus, Ricciardi et al. [193] sought to identify emerging scientific, technological, and socio-political issues that are likely to affect how invasion processes and dynamics have to be studied and managed within the next 20 years. They presented issues that are relevant to a broad range of taxa, environments, and geographical regions. These diverse issues suggest an expanding interdisciplinary role for invasion science in biosecurity and ecosystem management, burgeoning applications of biotechnology in alien species detection and control, and new frontiers, with particular attention to the microbial ecology of invasions. Scrutiny and debate that spurs the development of new research foci and policy objectives will be reinforced by such an approach [193].

Increasingly, the consequences of human-induced problems need to be considered fundamental on final ecosystem services and societal benefits, again focusing on both the ecological and economic repercussions of biopollution [194]. In short, biological invasions are an economic problem. Risks of invasions may be very low, but the potential damages are high [195,196,197]. Simpson [198] examines the economic side of the options facing the authorities wanting to control bioinvasions by applying the rules designed for emissions of traditional pollutants. It has to be underlined that the option of eradication, which has been applied in an early stage of introduction of marine populations of species known to be invasive, is very seldom applicable [199,200]. The marginal costs to achieve a reduction of risk by prevention or control, mitigation, and adaptation measures may appear disproportionate. Moreover, such costs are seldom reflected in market prices so that they are typically ignored. Control of potentially invasive species is sustained by public entities and consequently supported by the weakest components of society [195,201].

Considerations on these costs indicate a precautionary approach. The precautionary principle holds that where the effects of some activity are uncertain, but are potentially both costly and irreversible, society should take action to limit those effects before the uncertainty is resolved. The rationale for the principle is generally that the conjectured costs of not taking action are much greater than the known costs of preventative or anticipatory action [202]. This approach entails careful and well-organised communication [203] to the public and the notion that only individual and social behaviour changes [204] can succeed in obtaining the desired results in mitigating the threat by bioinvasions.

In response to the threats from invasive species, many countries have adopted the twin goals of preventing the introduction of new invaders and of controlling the spread and population size of those already established [205,206]. These policy goals have been formalized through local, state, and national legislation, and through the ratification of international treaties, such as the international Convention on Biological Diversity (CBD). Cost-effective solutions have been proposed involving also industrial counterparts [207].

In conclusion, biological pollution should be treated in the same way as other types of pollution, such as oil spills, and contingency plans for marine NIS introductions should be developed, including an efficient rapid response upon finding a newly introduced species, where possible (see [51,208]).

The scientific evidence gathered and summarized in this review paper strongly suggests maintaining a precautionary approach towards the intended and unintended human-aided introductions of species or their propagules into new areas. It is necessary to take into account not only the disruption of the dynamical ecological equilibria existing in the receiving environment [209], but also the risk of direct adverse effects. Such effects have been documented for a host of alien species that have proven to be detrimental to the economy, health, or in general to the wellbeing of human populations [4]. This is particularly the case for pathogens, venomous and toxic species [210,211], and parasites, which can be dangerous also because they are not known in the receiving areas, or in the case of fouling organisms, causing adverse effects to marine infrastructures and their functioning.

## Figures and Tables

**Table 1:** Examples of data bases containing information on introduced species at the global and regional level.

Data Base	Web Address
CABI Invasive Species Compendium	http://www.cabi.org/isc/ (accessed on 4 April 2021)
GISD-Global Invasive Species Database	http://issg.org/database/welcome/aboutGISD.asp (accessed on 4 April 2021)
WRiMS-World Register of Introduced Marine Species	http://www.marinespecies.org/introduced/ (accessed on 4 April 2021)
EASIN-European Alien Species Information Network	https://easin.jrc.ec.europa.eu/easin (accessed on 4 April 2021)
AquaNIS-Information system on aquatic non-indigenous and cryptogenic species	http://www.corpi.ku.lt/databases/index.php/aquanis/ (accessed on 4 April 2021)
NOBANIS European Network on Invasive Alien Species	https://www.nobanis.org/about-nobanis/ (accessed on 4 April 2021)
Invasive species in Belgium	http://ias.biodiversity.be/ (accessed on 4 April 2021)

**Table 2:** Comparison of the chemical and biological pollution characteristics.

Issues	Chemical Pollution	Biological Pollution
Types of pollution	Legacy pollutants	Established NIS
	Newly emerging man-made compound (xenobiotics)	New introductions
	Anthropogenic forcing in the increase of natural chemical substances	Biological invasions and spread
Detection and assessment methods	Chemical analysis of a limited set of elements/compounds in selected matrices	Rate of NIS introductions
	Comparison of levels found in pristine areas	Relative dominance of NIS vs. native species
	Toxicity on marine organisms	Extinction of native species
	Alteration of ecosystem functioning	Structural and functional ecosystem disruption
